# Compensation of Mutation in *Arabidopsis glutathione transferase* (*AtGSTU*) Genes under Control or Salt Stress Conditions

**DOI:** 10.3390/ijms21072349

**Published:** 2020-03-28

**Authors:** Edit Horváth, Krisztina Bela, Ágnes Gallé, Riyazuddin Riyazuddin, Gábor Csomor, Dorottya Csenki, Jolán Csiszár

**Affiliations:** 1 Institute of Plant Biology, Biological Research Centre, Temesvári krt. 62., H-6726 Szeged, Hungary; horvathedo@gmail.com; 2 Department of Plant Biology, Faculty of Sciences and Informatics, University of Szeged, Közép fasor 52., H-6726 Szeged, Hungary; belakriszti88@gmail.com (K.B.); riyazkhan24992@gmail.com (R.R.); tsomyer@gmail.com (G.C.); csenkidorottyazsuzsanna@gmail.com (D.C.); 3 Doctoral School in Biology, Faculty of Science and Informatics, University of Szeged, H-6726 Szeged, Hungary

**Keywords:** antioxidant enzyme activity, glutathione, glutathione reductase, glutathione transferase, gene expression, NaCl, redox state

## Abstract

Glutathione transferases (GSTs) play a crucial role in detoxification processes due to the fact of their glutathione (GSH) conjugating activity, and through glutathione peroxidase or dehydroascorbate reductase (DHAR) activities, they influence the redox state of GSH and ascorbate (AsA). The plant-specific tau (GSTU) group is the largest class of *Arabidopsis* GSTs, and their members are involved in responses to different abiotic stresses. We investigated the effect of salt stress on two-week-old *Arabidopsis* *thaliana* wild-type (Col-0), *Atgstu19* and *Atgstu24* mutant plants after applying 150 mM NaCl for two days. The *Atgstu19* seedlings had lower GST activity and vitality both under control conditions and after salt stress than the wild-type, but the level of total ROS was similar to the Col-0 plants. The GST activity of the knockout *Atgstu24* mutant was even higher under control conditions compared to the Col-0 plants, while the ROS level and its vitality did not differ significantly from the wild-type. Analysis of the *AtGSTU* expression pattern revealed that the mutation in a single *AtGSTU* gene was accompanied by the up- and downregulation of several other *AtGSTUs*. Moreover, elevated AsA and GSH levels, an altered GSH redox potential and increased DHAR and glutathione reductase activities could help to compensate for the mutation of *AtGSTU* genes. The observed changes in the mutants suggest that the investigated isoenzymes influence the redox homeostasis under control conditions and after NaCl treatment in *Arabidopsis* seedlings. These data indicate for the first time the more general role of a temporary shift of redox status as part of GST mechanisms and regulation.

## 1. Introduction

Glutathione transferases (GSTs) belong to a very ancient protein superfamily that is thought to have evolved in response to the development of oxidative stress. Plant genomes contain dozens of *GST* genes and most of the proteins can be found in homodimer or heterodimer form, leading to enormous diversity within GST protein families [1]. Glutathione transferases function both as catalytic enzymes with activity towards various substrates as well as non-catalytic, binding and carrier proteins [2,3]. Plant GSTs are grouped into ten classes and among them, the most abundant tau (GSTU), phi (GSTF), dehydroascrobate reductase (DHAR) and lambda (GSTL) isoenzymes are plant-specific. Dehydroascrobate reductase and GSTL enzymes catalyse redox reactions and participate in the recycling of antioxidants, such as ascorbate (AsA) and flavonols [4]. Tau and phi GSTs are involved mainly in xenobiotic metabolism, which may be related to their high affinity toward a broad spectrum of harmful compounds, including xenobiotics and endogenous stress metabolites. They not only have detectable glutathione-conjugating activity towards standard xenobiotic GST substrates, but via their glutathione peroxidase (GPOX) activity, they may take part in reducing the levels of H_2_O_2_ and other peroxides [5]. Moreover, by using glutathione (GSH, γ-Glu-Cys-Gly) as a co-substrate, their catalytic activity may reduce the pool of this redox-active molecule.

It is widely accepted that GSTs orchestrate and fine tune plant adaptation and tolerance to environmental stresses, pathogenic attackers and the detoxification of toxic chemicals and metabolites [2,3,6,7,8,9,10,11,12,13,14,15,16]. Conducting genetic screens to identify components of salt stress tolerance in *Arabidopsis* revealed that *AtGSTU19* and *AtGSTF9* can be important cytoplasmic proteins in salt stress responses [17]. During the investigation of the hardening mechanism, we previously found that the maintained or even increased GST and GPOX activities are important for efficient adaptation to the subsequent salt stress [11,18]. Among the investigated *Arabidopsis GST* genes, induction of *AtGSTU19* and *AtGSTU24* genes and elevated GST activity was observed in the leaves and roots of hydroponically grown eight-week-old plants after one week of 100 mM NaCl treatment [11].

In *Arabidopsis*, GSTUs include 28 members which can be the result of gene duplication events [19]. Tau class *Arabidopsis* GSTs can be divided into three separate clades using phylogenetic methods [5], but the importance of this differentiation is still unknown. Several studies describe the role of GSTUs in detoxification processes [20,21,22] and plants overexpressing *GSTU* genes have enhanced stress resistance [23,24,25]. Almost all of the *Arabidopsis* GSTUs have been found to selectively bind fatty acid derivatives and some of the isoenzymes are induced by abiotic stresses or play a role in signalling [2]. Under control conditions, AtGSTU19 represents a significant percentage of the GST pool [26]. Analysis of the expression pattern of *AtGST* genes based on micro-array data and using Genevestigator meta-analyser revealed that *AtGSTU19* was expressed in almost all of the investigated tissues and had the highest relative transcription level among the *AtGSTs* [19]. Transgenic plants overexpressing the *AtGSTU19* gene had enhanced salt-, drought- or methyl viologen stress tolerance, and it was suggested that AtGSTU19 may act as a stress regulator by increasing the activities of antioxidant enzymes to strengthen the ROS scavenging activity or maintain ROS homeostasis [27]. Comparing the effect of 75 and 150 mM NaCl treatments on the roots of seven-day-old knockdown *Atgstu19* mutants to the Col-0 wild-type, we previously found that the *Atgstu19* roots were more sensitive to the salt stress than the wild-type. *Atgstu19* had lower vitality, a higher amount of H_2_O_2_ and application of the redox sensitive green fluorescent protein (roGFP2) [28] affirmed a more oxidized redox state in all root zones compared to the Col-0 roots after 3 h (h) of treatment [29].

Redox homeostasis is a fundamental cell property in which regulation includes the control of reactive oxygen species (ROS) generation, sensing and readjustment of the cellular redox state. It is widely accepted that the ratio of reduced and oxidized glutathione (GSH:GSSG) is an effective marker of cellular redox homeostasis [30,31]. From the concentration of GSH and GSSG, the glutathione half-cell reduction potential (*E*_GSSG/2GSH_) can be calculated [32]. A great deal of evidence underpins the importance of ROS and redox homeostasis in the acclimation of plants to salinity (reviewed in Reference [33]), and the salt tolerance in some plants appears to correlate with their ability to scavenge ROS [11,18,34,35,36,37]. In order to keep ROS levels tightly regulated, different non-enzymatic antioxidants and enzymatic systems have evolved in aerobic organisms [38,39]. Non-enzymatic scavengers of ROS include AsA, GSH, phenolic compounds, carotenoids, flavonoids and tocopherol. Enzymatic ROS scavengers include superoxide dismutase (SOD), catalase (CAT), ascorbate peroxidase (APX), glutathione peroxidase-like (GPXL), DHAR and GST enzymes [40]. 

Although there is some information in the literature on the involvement of individual *AtGSTUs* in the salt stress responses of plants, their role is still poorly understood. Much evidence indicates that GSTUs significantly contribute to adaptation and have a positive role in tolerance to salinity and other abiotic environmental stresses [6,11,18,21,39,41]. On the other hand, AtGSTU17 was reported to be a negative component of the stress-mediated signal transduction pathway in adaptive responses to drought and salt stress [42]. Measuring the enzyme activities of bacterially expressed recombinant *Arabidopsis* GSTs assayed with 1-chloro-2,4-dinitrobenzene (CDNB), benzyl isothiocyanate and cumene hydroperoxide (CHP) model substrates suggested that related sequences have broadly similar enzyme activity spectrums, and it is more likely that there is a substantial overlap of activities and functional redundancy within the superfamily [5].

In the present experiments, we aimed to investigate the particular role of AtGSTU19 and AtGSTU24 in salt stress responses induced by 150 mM NaCl using T-DNA insertion mutants. While the two isoenzymes show high amino acid identity [5] and our earlier results indicated that both of them play positive roles in salt stress responses, the changes in total extractable GST activity in the two mutants showed different tendencies. The *Atgstu19* mutants had lower GST activity and vitality and higher dehydroascorbate (DHA) content than the other two investigated genotypes under control conditions, but the decreased ROS levels and maintained redox status indicate a successful induction of the antioxidant mechanisms. Here, we demonstrate that besides the altered *AtGSTUs* expression pattern, the changes in the redox-active antioxidant mechanism may contribute to the compensation for the effect of a mutation in an *AtGSTU* gene. The elevated DHAR and glutathione reductase (GR) activities in the untreated *Atgstu24* seedlings suggest that AtGSTU24 may also contribute to the regulation of redox homeostasis. These observed physiological and molecular changes highlight the importance of the redox-related processes as common part of the mechanisms of GSTUs.

## 2. Results

### 2.1. The Atgstu19 Mutant had Lower GST Activity, While the Atgstu24 Mutant had Higher Activity, in Comparison to the Wild-Type 

**Figure 1:**
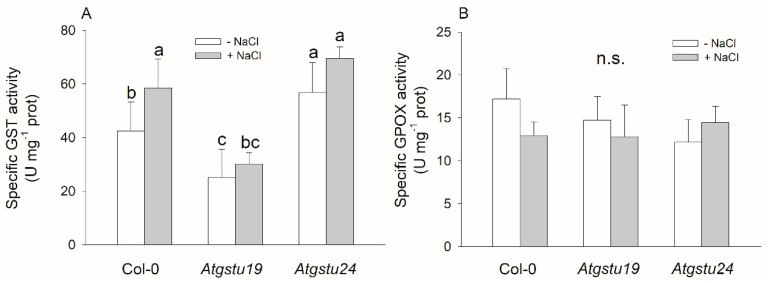
Main glutathione transferase activities of two-week-old wild-type (Col-0) and *GSTU* mutant (*Atgstu19* and *Atgstu24*) *Arabidopsis* plants treated with 150 mM NaCl for 48 h. (**A**) Glutathione transferase (GST) activity, (**B**) glutathione peroxidase (GPOX) activity of seedlings. Means ± SD denoted by different letters indicate a significant difference between the treatments at a *p* < 0.05 level according to Duncan’s test.

The 16 day old mutant lines did not show marked changes in their phenotype in comparison to the wild-type plants either in optimal conditions or under salt stress triggered by 150 mM NaCl. Measuring GST activity using the CDNB substrate revealed that knockdown mutation of the *AtGSTU19* gene resulted in decreased GST activity (38% lower than in wild-type controls) in seedlings under control conditions, in contrast to the mutation of *AtGSTU24,* in which the total GST activity was even higher (by 35%) than that of Col-0 plants. Treatment of plants with 150 mM NaCl for 48 h elevated the GST activity in the wild-type and *Atgstu24* lines (136% and 166%, respectively), but not in *Atgstu19* plants (Figure 1A). Glutathione peroxidase activity, measured with CHP substrate, did not differ significantly among the genotypes either under control conditions or after the NaCl treatment (Figure 1B).

### 2.2. The AtGSTU Gene Expression Profiles Changed in the Mutants

**Figure 2:**
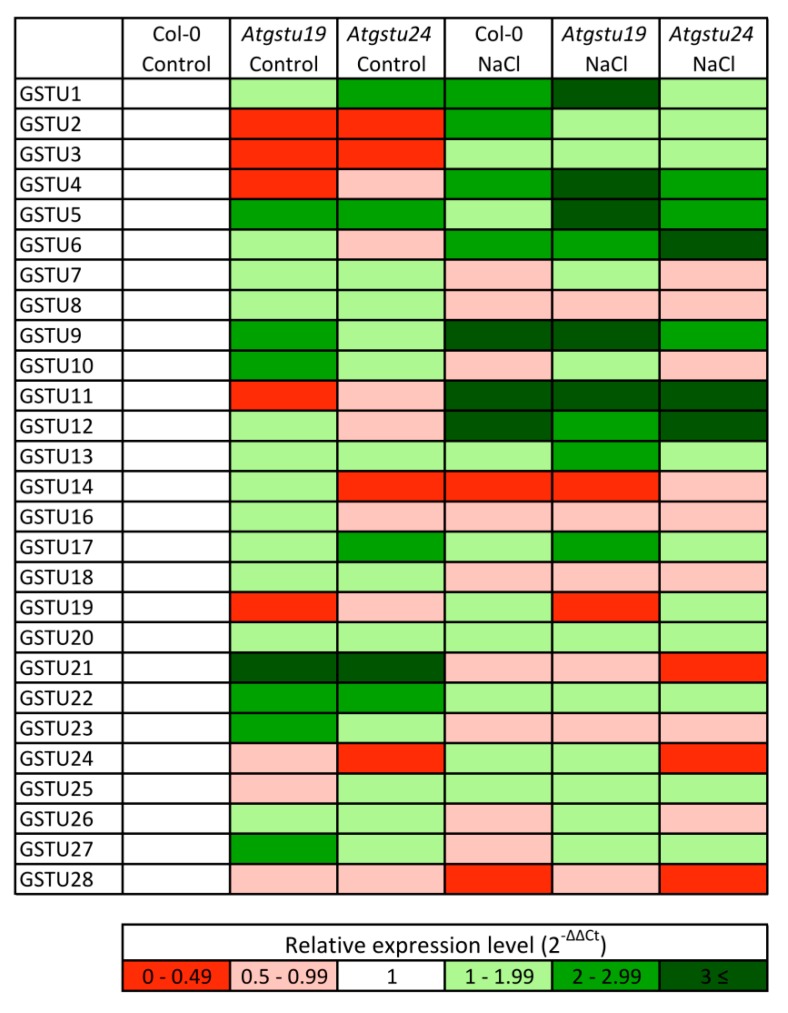
Heat map of 27 *A. thaliana* tau group glutathione transferase (*GSTU*) gene expression levels. Relative transcript amounts of *GSTU* genes were determined by RT-qPCR under control conditions (Control) and after 150 mM NaCl treatment (NaCl) in wild-type (Col-0) and *GSTU* mutant (*Atgstu19* and *Atgstu24*) *Arabidopsis* plants. For this, the expression of *GST* genes was normalized first by reference to the average transcript amount of *GAPDH2* and actin2 genes, and second to the average transcript amount of each *GST* genes in wild-type control plants. 2^-ΔΔCt^ data were presented as a heat map. Red colours show repression, while green colours show activation, according to the colour scale bar. The presented data are from one biological replicate.

Determination of the transcript amounts of the genes belonging to the tau class of GSTs by quantitative real-time PCR (RT-qPCR) revealed that *GSTU19* was expressed at the highest level among the *AtGSTUs* in the 16 day old Col-0 seedlings, while the expression level of *GSTU24* would be ranked eighth (2^-∆Ct^ data are shown in Table S1). To compare the expression pattern of *AtGSTUs* in the investigated genotypes, the transcript amount of each *AtGSTU* gene detected in Col-0 control plants was taken as an arbitrary one. The transcript amount of *AtGSTU19* in *Atgstu19* was approximately half of that in the wild-type, but that of *AtGSTU24* in the *Atgstu24* mutant was at the level of detectability. At the same time, the expression of several other *AtGSTU* genes was altered significantly in mutants (Figure 2, Table S1). Under control conditions, *AtGSTU1, AtGSTU5, AtGSTU21* and *AtGSTU22* were upregulated (2–6 times higher gene expressions than in Col-0) in both *Atgstu* mutants. Additionally, *AtGSTU9, AtGSTU10, AtGSTU23* and *AtGSTU27* were induced in the *Atgstu19* mutant, while in *Atgstu24*, *AtGSTU17* showed elevated expression compared to the wild-type. However, some genes such as *AtGSTU2* and *AtGSTU3* in both mutants, *AtGSTU4* and *AtGSTU11* in *Atgstu19* and *AtGSTU14* in *Atgstu24* showed repression.

Application of NaCl treatment to two-week-old seedlings induced the expression of *AtGSTU3-6, AtGSTU9, AtGSTU11* and *AtGSTU12* in all investigated lines after two days and *AtGSTU1* and *AtGSTU2* in Col-0 and *Atgstu19* plants. In *Atgstu19* mutants, *AtGSTU13* and *AtGSTU17* showed additional induction, furthermore the expression of the *AtGSTU5* gene was significantly higher than in Col-0 plants (Figure 2). Along with Col-0 plants’ repression of the *AtGSTU14* gene in *Atgstu19* and of the *AtGSTU28* gene in *Atgstu24*, *AtGSTU21* had lower expression only in *Atgstu24* mutants.

### 2.3. The Vitality and ROS Levels of Seedlings Indicated that the Mutants were Able to Cope with the Applied Salt Stress to Some Extent after 48 h

**Figure 3:**
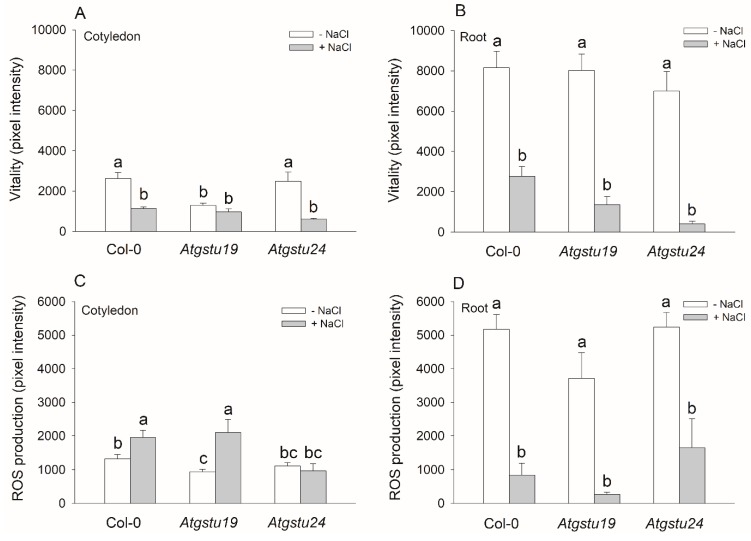
Cell vitality and level of reactive oxygen species (ROS) in cotyledons and root tips of two-week-old wild-type (Col-0) and *GSTU* mutant (*Atgstu19* and *Atgstu24*) *Arabidopsis* plants treated with 150 mM NaCl for 48 h. (**A**) Vitality of cotyledons and (**B**) root tips investigated by fluorescein diacetate. (**C**) ROS level in cotyledons detected by 2’-7’-dichlorodihydrofluorescein diacetate. (**D**) ROS level in root tips under the same conditions as described above. Means ± SE, *n* = 9 denoted by different letters indicate a significant difference between the treatments at a *p* < 0.05 level according to Duncan’s test.

The vitality and the level of ROS were investigated in the cotyledons and roots of 16 day old seedlings using fluorescent microscopy with fluorescein diacetate (FDA) and 2’-7’-dichlorodihydrofluorescein diacetate dyes, respectively. While in control conditions, there were no significant differences in the vitality and ROS levels between the wild-type and mutant roots, the FDA pixel intensity of *Atgstu19* cotyledons was about half of that in the wild-type, showing the lower vitality of this insertional mutant (Figure 3A,B). Interestingly, the ROS content was less in the *Atgstu19* cotyledons than in the Col-0 plants (Figure 3C). These parameters were used to assess the degree of stress after applying the salt treatment. The smallest decrease of vitality was in Col-0 plants and the highest was in *Atgstu24* cotyledons and roots after 150 mM NaCl treatment. The salt stress significantly enhanced the ROS content in the cotyledons of Col-0 and *Atgstu19* mutants, but in *Atgstu24* seedlings, it remained on the level of untreated controls. The ROS content of roots was decreased due to the salt stress in all investigated lines (Figure 3A–D). The MDA content of the mutants did not differ from that of the untreated or 150 mM NaCl-treated Col-0 plants, indicating that these mutants might successfully cope with the applied NaCl stress on a short-term scale (Table 1).

### 2.4. Elevated AsA and GSH Levels and Increased DHAR and GR Activities might Help to Compensate for the Mutation of AtGSTU Genes

**Figure 4:**
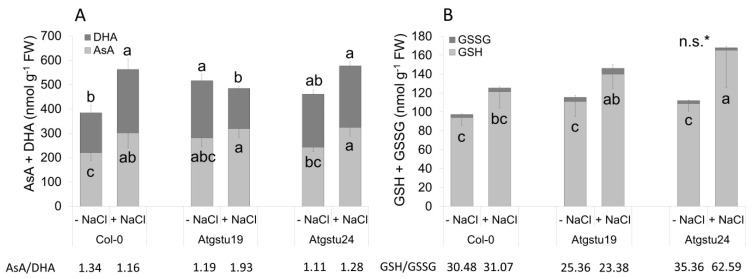
Ascorbate and glutathione contents of two-week-old wild-type (Col-0) and *GSTU* mutant (*Atgstu19* and *Atgstu24*) *Arabidopsis* plants treated with 150 mM NaCl for 48 h. (**A**) The reduced ascorbic acid (AsA) and the oxidized dehydroascorbate (DHA) contents in seedlings. (**B**) Reduced (GSH) and oxidized (GSSG) glutathione contents in seedlings. Means ± SD. Means denoted by different letters indicate a significant difference among the treatments at a *p* < 0.05 level according to Duncan’s test. * n.s.—not significant. There were no significant differences in GSSG content.

The total ascorbate content was higher in the *Atgstu19* and *Atsgtu24* mutants (by 34% and 19%, respectively) than in wild-type plants under control conditions (Figure S1). Application of 150 mM NaCl increased the total ascorbate level of Col-0 and *Atgstu24* mutant plants by 46% and 31%, respectively, while at the same time, the changes were not significant in the *Atgstu19* seedlings (Figure S1). Analysis of the ascorbate redox state revealed that in the untreated seedlings, the amount of the oxidized form of ascorbate (DHA) was elevated significantly in the *Atgstu19* lines, but after 48 h of salt treatment, the DHA content increased only in Col-0 and *Atgstu24* plants. Interestingly, the AsA/DHA ratio was lower in both *Atgstu* mutants than in Col-0 plants under control conditions, but it increased after salt treatment (Figure 4A).

The reduced glutathione (GSH) level of mutants was slightly enhanced compared to the wild-type seedlings under control conditions. The 150 mM NaCl treatment increased the glutathione content in all lines, but the increment was about 1.5—1.7-fold higher in mutant plants than in Col-0 (Figure S1). Although the oxidized glutathione (GSSG) contents of plants were not significantly different, the GSH/GSSG ratio was lower in *Atgstu19* plants than in Col-0 both under control and salt stress conditions. The salt treated *Atgstu24* seedlings had abundant amounts of total glutathione (GSH plus GSSG) and high GSH-to-GSSG ratios (Figure 4B). The calculated reduction potential of the GSH/GSSG couple became more negative due to the salt stress in all genotypes, but the highest changes (11.65 mV) were detected in the *Atgstu24* mutant (Table 2).

**Figure 5:**
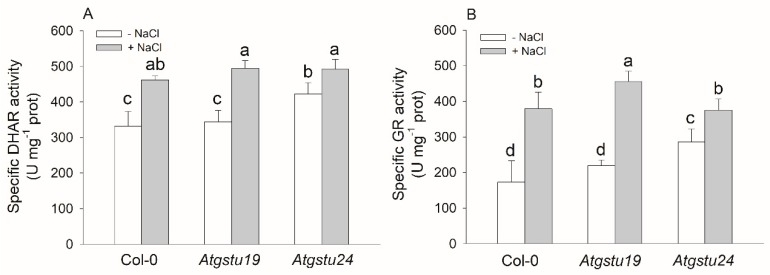
Redox-related antioxidant enzyme activities of two-week-old wild-type (Col-0) and *GSTU* mutant (*Atgstu19* and *Atgstu24*) *Arabidopsis* plants treated with 150 mM NaCl for 48 h. (**A**) Dehydroascorbate reductase (DHAR) activity of seedlings. (**B**) Glutathione reductase (GR) activity of plants. Means ± SD denoted by different letters indicate a significant difference between the treatments at a *p* < 0.05 level according to Duncan’s test.

Measuring the activity of some enzymes connected to the re-reduction AsA–GSH pool revealed that the *Atgstu24* mutant plants had the highest DHAR and GR activities and possessed the most negative redox potential among the untreated genotypes. The salt treatment elevated both the DHAR and GR activities in all investigated lines and the highest GR activity (approximately two-fold increment) was measured in the salt treated *Atgstu19* mutants (Figure 5).

## 3. Discussion

### 3.1. Arabidopsis Plants Compensated for the Decreased AtGSTU Transcripts with Altered AtGSTU Gene Expression and Elevated Antioxidant Activity

Glutathione transferases play a variety of functions in plant physiological processes under both normal and stress conditions. The members of the plant-specific tau GST class are stress inducible, have xenobiotic detoxification and glutathione peroxidase activities and play a role in the secondary metabolism during defence mechanisms [2,43]. 

Both AtGSTs investigated in this study, AtGSTU19 and AtGSTU24, belong to the third clade of the GSTUs, which consist of 10 members [2]. Their protein sequences show 71% amino acid identity (Figure S2) [44]. The main cis-regulatory elements found in the 5’ up-regulatory region of the genes are rather different, although both genes contain several presumably abiotic stress- and hormone-inducible sequences (Table S2). The AtGSTU19 represents a significant percentage of the GST pool in *Arabidopsis* [26], while AtGSTU24 came to the forefront especially according to its detoxification ability [9,45]. Both isoenzymes may have a role in salt stress responses and possess high activity toward the CDNB substrate [5,11,46]. The altered level of these proteins may significantly influence the total extractable GST activity of cells. Xu et al. [27] found that different transgenic lines overexpressing the *AtGSTU19* gene showed significantly enhanced GST activity toward CDNB compared to the wild-type.

In the search for clues to understand the role of AtGSTU19 and AtGSTU24 in defence against salt stress, we focused our attention on the changes after 48 h of the 150 mM NaCl treatment of two-week-old *A. thaliana* plants. Correlating well with the results of earlier studies [26], we found that knockdown mutation of the *AtGSTU19* gene resulted in decreased GST activity and vitality, both under control conditions and after applying 150 mM NaCl (49% in roots compared to salt-treated Col-0), but the level of total ROS was even lower than in the wild-type. The ROS level of the *Atgstu24* mutant did not differ significantly from that of the Col-0 plants, and its vitality decreased only in the presence of NaCl (53% in cotyledons and 14% in roots compared to salt-treated Col-0), but interestingly, the GST activity was even higher under control conditions than in the wild-type. The GPOX activity or the level of lipid peroxidation marker MDA were not significantly altered in the investigated genotypes.

Plant GSTs seem to have a high degree of functional overlap and variability both within and between classes [13]. The expression of particular GST isoenzymes in different plant organs and tissues and in response to different stimuli (e.g., constitutive versus stress-inducible) have been proposed as reasons to explain this apparent redundancy [47,48]. In our study, investigation of the *AtGSTU* transcript levels revealed that the expression of several *AtGSTU* genes was altered significantly in *Atgstu19* and *Atgstu24* mutants even under control conditions compared to the wild-type plants. Interestingly, four *AtGSTU* genes were induced and three were repressed in both mutants, respectively. Additionally, four other genes were induced in *Atgstu19*, while one gene in *Atgstu24* seedlings and one further gene in each *Atgstu* mutant was repressed. These changes in *GSTU* expression may explain the elevated GST activity in the *Atgstu24* mutant. It is possible that the induction of eight *GST* genes in the *Atgstu19* mutant was not sufficient to compensate for the lack of this extremely important GST isoenzyme concerning the total extractable glutathione S-transferase activity. Isoenzymes with an enhanced gene expression level in *Atgstu19* mutants were still expressed at a much lower level than *AtGSTU19* in the wild-type (Table S1); thus, their induction might not compensate for the missing activity. One other possible explanation for our results could be that the newly induced isoenzymes may have lower activity toward CDNB than AtGSTU19 [5,46]. 

Monitoring the known or predicted interactions of these isoenzymes in the STRING database [49] shed light on connections among the AtGSTs and DHARs (Figure S3 ). Dehydroascorbate reductases are responsible for the recycling of AsA and are also classified as belonging to the GST superfamily [2]. In our experiments, a single mutation of *AtGSTU* genes caused slightly increased total AsA in *Atgstu19* and *Atgstu24* seedlings (by 34% and 19%, respectively), but because of the elevated DHA content of mutants, the AsA/DHA ratio was lower. According to the known and predicted interactions, both AtGSTU isoenzymes have a close connection with AtDHAR1 and AtDHAR2 (first shell of interactors, Figure S3). Furthermore, this analysis highlighted that both AtGSTU19 and AtGSTU24 can closely interact with GR (responsible for the reduction of GSSG) and glutathione synthetase (GSH2) enzymes. Glutathione synthetase, which catalyses the second step of GSH biosynthesis (addition of glycine to γ-glutamyl-cysteine), is regulated by several factors [30].

The total GSH content in the untreated *Atgstu19* and *Atgstu24* mutants was enhanced by 18% and 13%, respectively (Figure S1). Rahantaniaina et al. [50] also found that *Atgstu24* mutant seedlings had slightly higher GSH content under control conditions. After 3-aminotriazole treatment, the lack of this isoenzyme did not induce the bleaching of the seedlings, suggesting that the plants can compensate for the mutation of *GSTU24* during oxidative stress [50]. In *Atgstu17* mutants, besides the elevated GSH levels, a higher GSH/GSSG ratio was measured [42,51]. In our previous experiments, we found that *Atgstf9* mutants also exhibited increased AsA and GSH pools, and their redox status was modified [10]. These results suggest that the increase in the GSH pool may be a common characteristic of *Atgst* mutants, at least for the members of tau and phi classes.

### 3.2. Is the Altered GSH Level of Mutants Needed to Cope With Salt Stress?

Constitutively high levels of reduced GSH are advantageous as they act as a strong buffer against ROS but would make the system less responsive to changes in redox potential that may be needed to upregulate the inducible defence components [52]. Proteomic analysis of *Arabidopsis* roots subjected to the 150 mM NaCl treatment revealed an increase in the amount of important ROS-scavenging and detoxifying proteins, including APX, glutathione peroxidase, Class III peroxidases, SOD and GSTs [53]. In the present study, we found that the salt stress was accompanied by enhanced DHAR and GR activities. Knockout *Atgstu24* also had higher DHAR activity and GSH content after salt treatment than wild-type plants and this could help to keep ROS under tight control and maintain a higher AsA/DHA and GSH/GSSG ratio. However, taking into account that the *Atgstu24* mutant had slightly lower vitality than the other two genotypes, it can be assumed that these changes were not suitable to allow the plant to cope successfully with salt stress. Although the lack of AtGSTU24 increased the total GST activity under control conditions and it was slightly elevated even after salt treatment, the induction of several *AtGSTU* genes (*AtGSTU3*-6, *AtGSTU9*, *AtGSTU11* and *AtGSTU12*) observed after salt treatment was in most cases lower than in *Atgstu19* or wild-type plants.

**Scheme 1:**
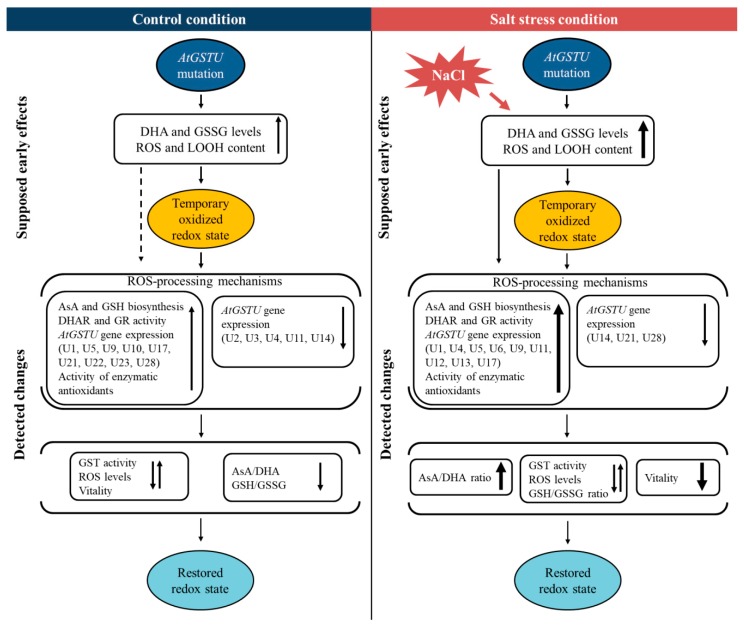
Schematic model summarizing the results presented in this study. Mutation in a tau group glutathione transferase (*AtGSTU*) gene supposedly induced the accumulation of ROS and lipid peroxides, which led to a temporary oxidized redox state within the cells accompanied by elevated dehydroascorbate and oxidized glutathione levels. To compensate these changes in *Atgstu* mutants the ascorbic acid and glutathione biosynthesis, the enzymatic antioxidant system (such as dehydroascorbate reductase and glutathione reductase activity) was induced and the *AtGSTU* gene expression pattern was modified. The alteration of the ROS-processing network might help to maintain the ROS and redox homeostasis in the mutants. Salt stress presumably intensified the accumulation of ROS, LOOH and oxidized non-enzymatic antioxidants leading to further enhancement of the AsA and GSH biosynthesis, the activity of antioxidant enzymes (DHAR and GR) and the expression of several *AtGSTU* genes. Although these changes helped *Atgstu* mutants to restore the redox potential after NaCl treatment, the vitality of mutants was lower than in wild-type. (An upward arrow indicates increase while a downward arrow shows decrease; the thickness of the arrows refers to the extent of the changes). Abbreviations: AsA, ascorbic acid; DHA, dehydroascorbate; DHAR, dehydroascorbate reductase; GSH, reduced glutathione; GR, glutathione reductase; GSSG, oxidized glutathione; GSTU, glutathione transferase tau group; LOOH, lipid peroxides; ROS, reactive oxygen species.

In the *Atgstu19* mutant, the GSH content was also increased, but the GSH to GSSG ratio was lower than in Col-0. Interestingly, the calculated redox potential from the measured data revealed similar redox status in this mutant than in the wild-type, both under control conditions and two days after application of the salt stress (Table 2). By analysing the redox status of *Arabidopsis* root tips using a roGFP1 redox sensor, Jiang et al. [54] demonstrated that the immersion of seedlings in 100 mM of NaCl for 3–24 h shifted the redox potential of the entire root toward the more oxidized status at the beginning, but it was re-established after 6 h and more negative redox potentials were detected compared to control roots, especially in the case of 24 h long treatment. Generally, the redox potential of roots depended on the strength and duration of the applied stress: while it might remain more negative in the case of mild (50 mM NaCl) stress, it became more oxidized in the presence of a higher (150 mM) salt concentration. However, after a few days, the salt-induced changes in redox potentials decreased and the differences in the redox status of seedlings practically disappeared [54]. Investigation of the redox status of five-day-old *Atgstu19* and *Atgstf8* mutants using a roGFP2 fluorescent probe revealed that the *Atgstu19* mutant had the most oxidized redox status in all root zones and under all investigated conditions [29]. Interestingly, the redox potential of the mutant roots showed smaller changes after applying 150 mM NaCl for 3 h than the wild-type, due to the fact of their already more positive value under control conditions. It was concluded that their increased salt sensitivity can be associated with the decreased redox potential response [29]. The lower GSH/GSSG ratio and increased GSH level observed in several *GST* mutants and overexpressing lines [41,55] and references above) suggest that altering the redox homeostasis can be part of a general mode of action in the mechanisms of GSTs. The dynamic interaction of GSTs with other glutathione-related enzymes might be how temporary redox status changes allow the regulation of normal cellular physiology. A schematic model illustrating predicted physiological and regulatory events in *Atgstu* mutants resulting in the observed changes in metabolite concentrations and enzyme activities under control conditions and after applying salt stress are summarized in Scheme 1.

## 4. Materials and Methods

### 4.1. Plant Material and Growth Conditions

Fourteen-day-old *Arabidopsis thaliana* (L.) Heynh. ecotype Columbia (Col-0) as a wild-type, *Atgstu19* and *Atgstu24* mutant seedlings were used in all experiments. *Arabidopsis* lines (SALK_041942 and SALK_034472) containing a T-DNA insertion in *AtGSTU19* (*At1g78380*) and *AtGSTU24* (*At1g17170*) genes were obtained from the Salk Institute [56] and the seeds of homozygous genotypes were used. The positions of T-DNA insertions and gene-specific PCR primers used for testing the segregation of T-DNA insertions are provided in the Figure S4. The *Atgstu19* is a knockdown mutant (the transcript amount of *AtGSTU19* was about half of that in the wild-type), while the *Atgstu24* can be regarded as knockout mutant (Table S1). Stress treatments were carried out on two-week-old in vitro grown plants by placing them onto agar-solidified culture medium supplemented with 150 mM NaCl. The Petri dishes were kept in growth chamber (Fitoclima S 600 PLH, Aralab, Portugal) at a photon flux density of 100 μmol m^−2^ s^−1^ (10/14 day/night period), at a relative humidity of 70% and 21 °C. Samples were collected after 48 h NaCl treatment. The experiments were repeated at least two times, the measurements were performed in three replicates unless indicated otherwise.

### 4.2. Determination of Glutathione Transferase and Glutathione Peroxidase Enzyme Activities

To analyse the enzyme activity, 200 mg tissue was homogenized on ice in 1 mL 100 mM phosphate buffer (pH 7.0) containing 1 mM phenylmethylsulfonyl fluoride and 1% polyvinyl-polypirrolidone. The homogenate was centrifuged for 20 min at 10,000× *g* at 4 °C, and the supernatant was used for enzyme activity assays. Uvikon 930 spectrophotometer (Kontron AG, Eching, Germany) was used for every absorption measurement in our experiments

Glutathione transferase (GST, EC 2.5.1.18) activity was determined as published earlier [11] with some modifications. The GST activity was determined spectrophotometrically using an artificial substrate, 1-chloro-2,4-dinitrobenzene (CDNB, Sigma–Aldrich, Germany). The GST activity was given in specific activity (μmol conjugated products min^−1^, ε_340_ = 9.6 mM^−1^ cm^−1^).

Glutathione peroxidase (GPOX, EC 1.11.1.9) activity was measured with cumene hydroperoxide (CHP; Sigma–Aldrich, Germany) as a substrate. The reaction mixture contained 4 mM GSH, 0.2 mM NADPH, 0.05 U of GR (from baker’s yeast, Sigma–Aldrich, Germany), 100 µL enzyme extract, and 0.5 mM substrate in phosphate buffer (0.1 M, pH 7.0) in a total volume of 1 mL. The GPOX activity was given in specific activity (μmol of converted NADPH min^−1^, ε_340_ = 6.22 mM^–1^ cm^–1^).

### 4.3. Determination of Glutathione Reductase and Dehydrosacorbate Reductase Activities

Dehydroascorbate reductase (DHAR, EC 1.8.5.1) and glutathione reductase (GR, EC 1.8.1.7) activities were determined as described at GST and GPOX activity determination, with the exception that 50 mM phosphate buffer was used for homogenization

The DHAR activity was assayed as was published in Csiszár et al. [18]. The enzyme activity (nmol min^−1^ mg^−1^ protein) was calculated from the increase in the AsA content by measuring the absorbance at 265 nm and using the 14.0 mM^−1^ cm^−1^ extinction coefficient.

The GR activity was determined by measuring the absorbance increment at 412 nm when 5,5’-dithio-bis(2-nitrobenzoic acid) (DTNB) was reduced by GSH, generated from GSSG [40]. The activity was calculated as the amount of reduced DTNB, in nmol min^–1^ mg^−1^ protein, ε_420_ = 13.6 mM^–1^cm^–1^. 

### 4.4. RNA Purification and Expression Analyses with Quantitative Real-Time PCR

The expression rate of *Arabidopsis* GST genes was determined by quantitative real-time PCR (RT-qPCR) after the purification of RNA from 100 mg plant material according to Chomczynski and Sacchi [57] as was described in Bela et al. [40]. The primers used for the RT-qPCR are given in Table S3. The expression rate of *GST* genes was monitored as published earlier in Bela et al. [40]. Data analysis was performed using qTOWER Software 2.2 (Analytik Jena, Jena, Germany) software. The GAPDH2 (*At1g13440*) and actin2 (*At3g18780*) genes were used as internal controls, respectively [17]. The average Ct number of *GAPDH2* and actin2 gene was used for data normalization. Data of RT-qPCR was calculated using the 2^(-∆∆Ct)^ formula [58]. To demonstrate the differences between changes in the expression levels of the investigated GST genes, the relative transcript level in the *Arabidopsis thaliana* Col-0 control root samples were considered to be one for each gene, and the results were presented on a heat map created in Microsoft Excel using the conditional formatting function [40]. To demonstrate the differences in the enhancement of gene expression levels a lighter green colour indicates if the increase was between 2 - 2.99 and dark green if more than 3 fold elevation occurred compared to the level in the wild-type control.

### 4.5. Investigation of Vitality and Reactive Oxygen Species (ROS) using Fluorescent Microscopy

Zeiss Axiowert 200M microscope (Carl Zeiss Inc., Jena, Germany) equipped with a high-resolution digital camera (Axiocam HR, HQ CCD camera) and suitable filter sets was used for the fluorescence detection of vitality and ROS in roots and cotyledons of *Arabidopsis* plants. Fluorescein diacetate (FDA) was applied for the determination of cell vitality according to Horváth et al. [29]. To detect ROS production, 2’-7’-dichlorodihydrofluorescein diacetate (Sigma-Aldrich, Germany) was used according to Horváth et al. [11]. The intensity of fluorescence was measured on digital images with the help of Axiovision Rel. 4.8 software (Carl Zeiss Inc., Jena, Germany). Fluorescence intensity values were determined in 80 μm diameter circles, at 150 μm distance from the root tip and in 300 μm diameter circles in cotyledons. The diameter of circles was not modified during the experiments. The measurements were performed in 10 replicates.

### 4.6. Determination of Malondialdehyde Content

Malondialdehyde (MDA) formation was followed by using the thiobarbituric acid method [11]. 50 mg tissue was homogenized with 500 μL, 0.1% trichloroacetic acid (TCA) and 50 μL 4% butylhydroxytoluene was added to avoid further lipid peroxidation. The absorbance was measured at 532 nm and adjusted for non-specific absorbance at 600 nm. MDA concentration was calculated using an extinction coefficient of 155 mM^−1^ cm^−1^.

### 4.7. Ascorbate and Glutathione Extraction and Determination

Ascorbate and glutathione contents were determined according to Bela et al. [40]. Two-hundred mg of seedlings was homogenized with 0.8 mL of 5% TCA. The homogenate was centrifuged at 10,000× *g* for 20 min at 4 °C, and the supernatant was used for further determinations. To assay total ascorbate (AsA), 100 μL of 10 mM dithiothreitol (DTT) was added to the extract and the excess of DTT was removed by adding 100 μL, 0.5% (w:v) N-ethylmaleimide (NEM). Ascorbate concentrations were determined spectrophotometrically at 525 nm. Dehydroascorbate (DHA) content was calculated as the difference between the concentration of total and reduced ascorbate.

Total and oxidized glutathione concentrations were measured spectrophotometrically using an enzymatic assay. To mask reduced glutathione (GSH) 4-vinylpyridine was added to the extract and incubated for 60 min. The reaction mixture contained 0.1 M phosphate buffer pH 7.5, 1 mM 5,5′-dithio*bis*(2-nitrobenzoic acid) (DTNB), 1 mM NADPH, 1 U of glutathione reductase (GR baker’s yeast, Sigma-Aldrich, Germany) and 20 μL of the tissue extract in 1 mL volume. The GSH content was calculated from the difference between the concentration of total and oxidized glutathione. Standard curves were obtained for total glutathione and GSSG within the 0-2 μM range.

The reduction potential of the GSH/GSSG couple (the half-cell redox potential; E_hc_) was determined with the Nernst equation using the formula of Schafer and Buettner [32]: E_hc_= -240 - (59.1/2) log([GSH]^2^/[GSSG]) mV; where -240 mV is the standard reduction potential of glutathione on 25°C, pH 7.0 [59].

### 4.8. Statistical Analysis

Statistical analysis was carried out with SigmaPlot 11.0 software (SigmaPlot, Milano, Italy) by Duncan’s test and differences were considered significant at *p* ≤ 0.05. Data presented here are the means ± SD of at least 3 measurements unless indicated otherwise.

## 5. Conclusions

Here we have shown that mutation in a single *AtGSTU* gene not only caused changes in the expression of *AtGSTUs* but altered the redox homeostasis of the seedlings too. The two investigated mutations (i.e., Atgstu19 and Atgstu24) triggered different responses in many aspects in *Arabidopsis* seedlings, but, at the same time, the redox-related processes are common elements of them. According to our results, the knockdown *Atgstu19* mutants decreased GST activity which did not increase after salt treatment despite of the increased expression of eight *AtGSTU* genes. These results provide the first evidence that AtGSTU19 is irreplaceable in seedlings. However, these plants had elevated GSH content and GR activity to compensate for 48-h long NaCl treatment-induced changes. Interestingly, in contrast to *Atgstu19*, *Atgstu24* had elevated GST, DHAR and GR activities and AsA and GSH levels both under control conditions and after salt treatment. Maintained or even increased GSH/GSSG ratios could support the preservation of the redox potential of plant cells during salt stress in mutants, but it may also influence their response. As far as we know, this is the first report which demonstrate that mutation of AtGSTU genes with similar function act differently on the total extractable GST activity. Moreover, we propose that the redox-coupled changes are common components of the mechanisms of GSTs. On the one hand, our results underline that highly similar GST isoenzymes have a unique role and regulation in plants. On the other hand, altered GSH and AsA levels as well as the *GSTU* gene expression pattern in *Atgstu* mutants suggest that the investigated isoenzymes influence redox homeostasis under control conditions and after salt treatment in *Arabidopsis* seedlings.

## Figures and Tables

**Table 1:** Malondialdehyde (MDA) content of two-week-old wild-type (Col-0) and glutathione transferase (*GSTU*) mutant (*Atgstu19* and *Atgstu24*) *Arabidopsis* plants treated with 150 mM NaCl for 48 h.

	MDA content (nmol g^−1^ FW)	
	- NaCl	+NaCl
Col-0	17.66 ± 1.14 ^n.s.^	19.43 ± 1.83 ^n.s.^
*Atgstu19*	20.22 ± 2.89 ^n.s.^	16.11 ± 3.08 ^n.s.^
*Atgstu24*	15.38 ± 0.68 ^n.s.^	17.44 ± 1.35 ^n.s.^

Means ± SD; n.s.—not significant at a *p* < 0.05 level according to Duncan’s test.

**Table 2:** The reduction potential of the GSH/GSSG couple (half-cell reduction potential; E_hc_) determined with the Nernst equation in two-week-old wild-type (Col-0) and *GSTU* mutant (*Atgstu19* and *Atgstu24*) *Arabidopsis* plants treated with 150 mM NaCl for 48 h.

	E_hc_ (mV)	
	- NaCl	+NaCl
Col-0	−252.32 ± 6.16 ^n.s.^	-256.73 ± 10.28 ^n.s.^
*Atgstu19*	−252.27 ± 3.75 ^n.s.^	-255.32 ± 11.77 ^n.s.^
*Atgstu24*	−257.46 ± 3.45 ^n.s.^	-269.11 ± 8.96 ^n.s.^

Means ± SD; n.s.— not significant at a *p* < 0.05 level according to Duncan’s test.

## Supplementary Materials

The following are available online at https://www.mdpi.com/1422-0067/21/7/2349/s1, Figure S1: total ascorbic acid (AsA) and glutathione (GSSG) content of seedlings; Figure S2: amino acid identity of AtGSTU19 and AtGSTU24 isoenzymes; Figure S3: protein–protein interactions according to STRING; Figure S4: insertion site and position of primers used in genotyping of *AtGSTU* mutants; Table S1: 2^-ΔCt^ values of investigated *AtGSTU* genes; Table S2: 5’-regulatory elements of *AtGSTU19* and *AtGSTU24* genes; Table S3: primers used in RT-qPCR.
